# LINC00473 as an Immediate Early Gene under the Control of the EGR1 Transcription Factor

**DOI:** 10.3390/ncrna6040046

**Published:** 2020-11-12

**Authors:** Vincenza Aliperti, Emilia Vitale, Francesco Aniello, Aldo Donizetti

**Affiliations:** 1 Department of Biology, University of Naples Federico II, 80126 Naples, Italy; vincenza.aliperti@unina.it (V.A.); faniello@unina.it (F.A.); 2 NeurOmics Laboratory, Institute of Biochemistry and Cell Biology (IBBC), CNR, 80131 Naples, Italy; emilia.vitale@cnr.it

**Keywords:** immediate-early genes, long non-coding RNAs, LINC00473, C6orf176, EGR1, BDNF

## Abstract

Immediate early genes play an essential role in cellular responses to different stimuli. Many of them are transcription factors that regulate the secondary response gene expression. Non-coding RNAs may also be involved in this regulatory cascade. In fact, they are emerging as key actors of gene expression regulation, and evidence suggests that their dysregulation may underly pathological states. We previously took a snapshot of both coding and long non-coding RNAs differentially expressed in neuronal cells after brain-derived neurotrophic factor stimulation. Among these, the transcription factor EGR1 (a well-known immediate early gene) and LINC00473 (a primate-specific long non-coding RNA) that has emerged as an interesting RNA candidate involved in neuronal function and in cancer. In this work, we demonstrated that LINC00473 gene expression kinetics resembled that of immediate early genes in SH-SY5Y and HEK293T cells under different cell stimulation conditions. Moreover, we showed that the expression of LINC00473 is under the control of the transcription factor EGR1, providing evidence for an interesting functional relationship in neuron function.

## 1. Introduction

Immediate early genes (IEGs) are rapidly and transiently upregulated in response to different types of extracellular stimuli [1,2]. Their induction does not require de novo protein synthesis and commonly occurs within a few hours after the stimulation event [1]. Many induced IEGs, such as c-FOS, ARC, and EGR1, are protein-coding genes that function as transcription factors triggering the expression of the secondary response genes [3]. Recently, non-coding RNAs (ncRNAs), including microRNAs (miRNAs) and long ncRNAs (lncRNAs), have also been included in the molecular mechanisms underlying the gene expression regulation in the initial phase of the stimulus-induced molecular cascade [2,4,5], although their role in the immediate early response is not well understood. Interestingly, many lncRNAs are stimulus-inducible and harbor RNA polymerase II (Pol II) paused in the promoter-proximal site [6,7], suggesting a critical checkpoint between the early and processive elongation of Pol II for lncRNA transcription. These data also emphasize the inducibility of lncRNA genes by transcriptional activators upstream and in the proximity of TSS for gene activation [6,8,9,10]. LncRNAs may play pivotal roles in gene expression, acting as decoys, scaffolds, or guides, by interacting with DNA, RNAs, or proteins [11,12,13,14]. Notably, there are more lncRNAs than protein-coding genes in the human genome [15], and many of them are brain specific [16,17] suggesting a key role for lncRNAs in the evolution of higher brain functions [18]. They have been demonstrated to be essential for brain development and higher cognitive abilities, and involved in psychiatric and neurodegenerative diseases [18,19,20,21,22,23,24,25,26,27,28,29,30,31,32].

LINC00473, also known as Chromosome 6 open reading frame 176 (C6orf176), is a primate-specific cAMP pathway-responsive lncRNA [33] that has been shown to be differentially expressed in the immediate response phase of BDNF (brain-derived neurotrophic factor) stimulation in a neuronal cell model [4]. LINC00473 is regulated by synaptic activity and has been hypothesized to control the gene expression kinetics in the immediate early response in human iPSC-derived neurons [34]. Recently, LINC00473 was also involved in depression in a sex- and brain site-specific manner [35]. In particular, Issler and colleagues [35] demonstrated its expression downregulation in the prefrontal cortex of depressed females with consequent selective changes in synaptic function and gene expression. The results obtained in both female mice and cultured human neuron-like cells showed LINC00473 as a CREB effector [35], emphasizing the role of the lncRNA as a female-specific driver of stress resilience that is aberrant in female depression [35].

In this study, we added further insights on the molecular mechanisms that regulate the expression of the lncRNA LINC00473. We first provided evidence that confirmed the IEG kinetics of the LINC00473 gene expression in two different neuronal cell model (SH-SY5Y and HEK293T). Second, we showed that this expression pattern could be under the control of EGR1 (early growth response 1), a well-known IEG involved in neuron function.

## 2. Results

**Figure 1:**
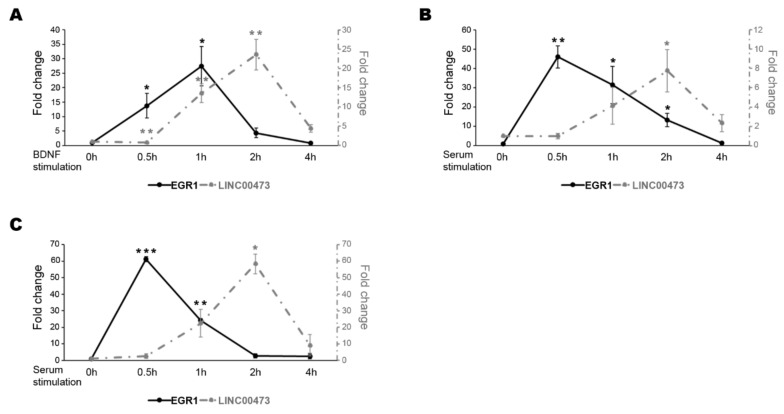
Expression pattern comparison of EGR1 and LINC00473 under BDNF stimulation. Expression level change in N-enriched SH-SY5Y cells after BDNF stimulation (**A**), in N-enriched SH-SY5Y cells after serum stimulation (**B**), and in HEK293T cells after serum stimulation (**C**) at the indicated time points for EGR1 (black line) and LINC00473 (dashed grey line). The sample at time 0 h was used as the calibrator. Gene expression levels were normalized to the reference gene Glyceraldehyde-3-phosphate dehydrogenase (GAPDH) and calculated by the 2^−ΔΔCt^ method. The results from independent biological replicates are expressed as mean of fold change ± SEM. Statistical analysis of the qPCR data was carried out using a two-tailed t-test. Significance of difference from time 0 h (* *p* ≤ 0.05, ** *p* ≤ 0.001, *** *p* ≤ 0.0001) is shown.

In our previous study [4], we showed that EGR1 and the lncRNA LINC00473 expression levels increased in 6-day treated retinoic acid (RA)-differentiated N-enriched SH-SY5Y cells after one hour (h) of BDNF stimulation [4]. Here, we extended the expression analysis in an extended time window after BDNF stimulation. As shown in Figure 1A, the EGR1 transcript level increased starting from 0.5 h, reached a peak after 1 h of BDNF treatment and then decreased with classical kinetics of the IEGs. Likewise, the LINC00473 expression resembles the IEGs reaching a peak after 2 h of treatment and then decreasing at 4 h (Figure 1A).

We also checked the expression pattern after serum stimulation in N-enriched SH-SY5Y. Serum stimulation is often used to study IEGs because serum response factors (SRF) mediate the signal-stimulated transcriptional induction of these genes by binding the serum response elements (SRE) in their promoters [36,37,38]. Even in this case, LINC00473 gene expression showed the typical kinetics of an IEG reaching a peak after 2 h of serum stimulation and then decreasing at 4 h. In addition, we showed that the expression of EGR1 anticipated that of LINC00473 corroborating the idea that EGR1 could be involved in the regulation of the LINC00473 transcription (Figure 1B). We extended the analysis to HEK293T cells that represent an easier cellular model for genetic manipulation. This model is often used for neuroscience-related studies. We also confirmed the previous results in this cellular system after different time points of FBS stimulation (Figure 1C).

To corroborate the hypothesis that EGR1 regulates the expression of LINC00473 under FBS stimulation, we planned a genome-editing approach based on CRISPR/Cas9 technology to obtain EGR1-knockout (KO) in N-enriched SH-SY5Y and HEK293T cells (Figure S1). The cells were transfected with the all-in-one plasmid pSpCas9(BB)-2A-Puro (PX459) V2.0, which includes both the coding region for Cas9 and the coding sequence for the gRNA. The gRNA was designed using an online tool reported by Ran and colleagues [39] to target the coding sequence just downstream of the start codon and obtain a frameshift mutation that affects the translation of a functional protein (Figure S1A). After the puromycin selection of positive transfected clones, we isolated and analysed a single cell-derived population. The sequence analysis of the PCR-amplified target genomic region showed that we obtained a population carrying one single nucleotide deletion (Figure S1B). The INDEL (INsertion/DELetion) determined the generation of a premature stop codon because of a frame-shift mutation (Figure S1B). To further validate the EGR1-KO cell lines, we performed western blotting to show the lack of functional protein production. The analysis suggested that the protein level strongly increased in wild-type (WT) cells after 1 h of serum stimulation while the corresponding protein was completely absent in the KO cells (Figure S1C,D).

**Figure 2:**
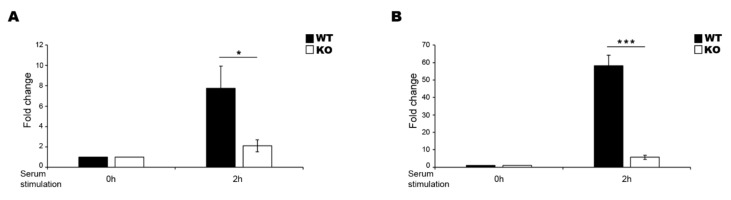
Effect of serum stimulation on EGR1 WT and KO cells. LINC00473 expression level change in N-enriched SH-SY5Y EGR1 WT and KO cells (**A**) and in HEK293T EGR1 WT and KO cells (**B**) after 2 h of serum stimulation. The sample at time 0 h was used as the calibrator. Gene expression level was normalized to the reference transcript GAPDH and calculated by the 2^−ΔΔCt^ method. The results from independent biological replicates are expressed as mean of fold change ± SEM. Significance of difference (* *p* ≤ 0.05, *** *p* ≤ 0.0001) is shown.

To test whether the EGR1-KO affected the LINC00473 expression, we analysed the lncRNA expression levels in WT and KO cells after 2 h of serum stimulation (Figure 2). LINC00473 expression was strongly affected in both N-enriched SH-SY5Y KO cells (Figure 2A) and HEK293T KO cells (Figure 2B), corroborating the idea that EGR1 is a transcription factor that regulates the LINC00473 expression in this experimental condition.

**Figure 3:**
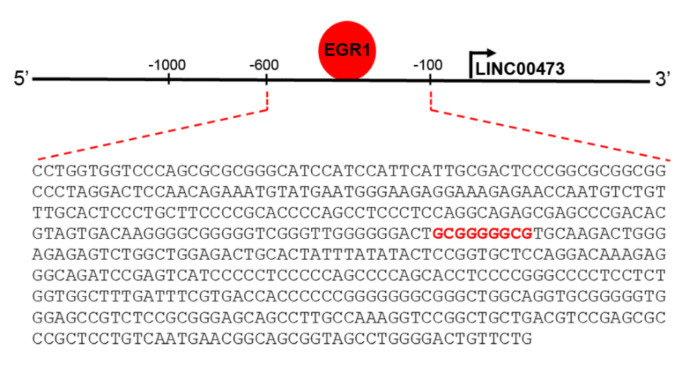
Schematic representation of LINC00473 promoter region showing the EGR1 binding site (GCGGGGGCG) at −393/−385 bp.

The transcription factor EGR1 is an IEG involved in either brain physiological and pathological conditions, most likely due to its involvement in critical processes controlling neuronal activity. EGR1 is involved with neurotransmission functions and synaptic plasticity, reaching to higher order neuronal processes such as learning and memory, response to emotional stress, and reward [40]. As an IEG, it is rapidly up-regulated in neurons following different stimuli. It orchestrates the subsequent gene expression to allow long-term neuronal response [4,36,40,41,42]. However, relatively little is known about the exact transcriptional regulatory programs of IEG, especially for those correlated with effectors involved in the early response following its activation. A good indicator of direct transcriptional EGR1 control is represented by the presence of specific EGR response element (GCGG/TGGGCG) [43,44]. Genome-wide techniques have opened the possibility to search for EGR1-regulated genes on a large scale, and several studies have investigated EGR1 binding through chromatin immunoprecipitation following by deep sequencing (ChIP-seq) [40,45]. The ENCODE Transcription Factor Targets Dataset (http://amp.pharm.mssm.edu/Harmonizome/gene_set/EGR1/ENCODE+Transcription+Factor+Targets [46]) reported that LINC00473 is an EGR1 target. In silico analysis to predict transcriptional factors binding to LINC00473 promoter region using PROMO (http://alggen.lsi.upc.es/cgi-bin/promo_v3/promo/promoinit.cgi?dirDB=TF_8.3 [47]) and Alibaba2.1 (http://gene-regulation.com/pub/programs/alibaba2/index.html? [48]) tools confirmed the presence of the specific EGR response element (GCGGGGGCG) at the level of the LINC00473 promoter (Figure 3). 

## 3. Discussion

The immediate-early response mediates a cell fate in response to a variety of extracellular stimuli. Cells respond to stimuli through a set of genes known as IEGs that are primed for rapid activation and then rapidly switched off [1]. IEGs are elements involved in many cellular processes including differentiation and proliferation, and often are dysregulated in pathological states and cancer where they become continuously active [49,50,51]. Many IEGs encode transcription factors that regulate secondary response genes [52]. This expression is delayed because they require de novo protein synthesis. However, the earliest transcriptional response involves ncRNAs [2,4,5]. They function as regulatory molecules in different cellular processes, and their expression is regulated by transcription and RNA processing/stability [5].

In our previous work [4], we used microarray technology to identify differentially expressed lncRNAs in an immediate response phase of BDNF stimulation in a neuronal cell model. This provides clear evidence of their involvement, as master regulators, in early gene expression cascade triggered by BDNF. We found LINC00473 (or C6orf176) as the most differentially expressed lncRNA after 1 h of BDNF stimulation [4]. LINC00473 has been already showed to act as an oncogene in cancers where it is constitutively up-regulated and it likely affects cell proliferation, colony formation, cellular invasion, and epithelial-mesenchymal transition (EMT) [53,54,55,56,57,58,59,60,61,62]. Here, we show an expression pattern that resembles the IEGs in both BDNF- and serum-stimulations. Our data agree with Reitmair and colleagues [33] who describe LINC00473 as a primate-specific IEG able to respond to the EP2 and EP4 agonists treatment. The expression pattern comparison of the EGR1 and LINC00473 genes after different stimulations in neuronal cells corroborate the idea that the LINC00473 gene can be regulated by the transcription factor EGR1. Similar results were also confirmed in HEK293T cells that are often used as a surrogate of neuronal cell models because of a neural crest origin. This explains the expression of several neuron-specific genes [63,64]. In particular, EGR1-KO affected the LINC00473 expression, suggesting a role for LINC00473 as a delayed primary response gene in the EGR1-dependent regulatory cascade. Indeed, the transcriptional program induced by stimulation also involves a large group of delayed primary response genes that differ from IEGs in both their functions and genomic architecture [52]. Both immediate early and delayed primary response genes were induced at the transcriptional level, but the second ones are characterized by a delay in transcription initiation or elongation [52]. The IEGs are enriched in molecular function terms related to transcriptional regulation while the delayed primary genes are not associated with transcriptional regulators, and may function as transiently effectors of the IEG transcriptional program inducing the secondary response gene expression [52]. We previously reported the analysis of genes constitutively expressed in EGR1-over-expressing HEK293T and noted that LINC00473 was not included in that genes likely because some molecular mechanism ensures that LINC00473 is only transiently expressed in that condition [65] highlighting that its normal kinetics is that of the IEGs. 

In the central nervous system, neuronal plasticity and neurotransmission require complex interactions between genes and environmental stimuli [66]. It is clear that the IEGs represent a key component of these interactions and provide rapid and dynamic response to neuronal activity through the expression regulation of secondary genes [36,42]. The transcription factor EGR1 is a major mediator and regulator of synaptic plasticity and neuronal activity in both physiological and pathological conditions [40]. In particular, in neuropsychiatric disorders, such as depression, anxiety, and schizophrenia, EGR1 levels are lower in female- and brain site-specific manner when compared to healthy controls [40,67,68,69]. Interestingly, the LINC00473 downregulation in the prefrontal cortex of depressed females [35] is in line with low EGR1 levels [40,67], corroborating our hypothesis that EGR1 may regulate the LINC00473 expression pattern in brain function and development. Overall, although the direct involvement of EGR1 in the transcriptional regulation of LINC00473 remains to be determined, our results documented an interesting relationship between the EGR1 and LINC00473 genes that can lay the foundation for future studies to investigate their interplay in neuron function.

## 4. Materials and Methods

### 4.1. Cell Culture

N-enriched SH-SY5Y (human neuroblastoma, ATCC^®^, Manassas, VA, USA) and HEK293T (human embryonic kidney, ATCC^®^) cell lines were grown and propagated in Dulbecco’s Modified Eagle’s Medium (DMEM, EuroClone^®^, Milan, Italy) supplemented with 2 mM L-glutamine (EuroClone^®^), a solution of 1% penicillin/streptomycin (EuroClone^®^), and 10% fetal bovine serum (FBS, EuroClone^®^). In particular, the N-enriched population of SH-SY5Y was obtained from the parental cell line by a procedure reported elsewhere [4].

### 4.2. Cellular Treatments

The N-enriched SH-SY5Y cells were differentiated by incubation in a low serum (1.5%) medium containing 10 µM RA (retinoic acid, SIGMA-Aldrich^®^, St. Louis, MO, USA). In particular, 6 × 10^5^ cells were seeded in 35-mm plates. Starting on the following day, RA was added, and the medium was refreshed every 2 days. Untreated cells were grown in the presence of only dimethyl sulfoxide (DMSO, SIGMA-Aldrich^®^) as a vehicle control. After 6 days of differentiation, the medium containing RA was removed and substituted with a medium without FBS and with 10 ng mL^−1^ BDNF (PeproTech^®^, London, UK) for up to 4 h.

For serum stimulation, 6 × 10^5^ N-enriched SH-SY5Y and HEK293T cells were seeded in 35-mm plates and starved for 24 h by reducing FBS to 1.5%. After the starvation, cells were stimulated with 10% and 15% FBS, respectively, and collected after different time intervals up to 4 h.

### 4.3. RNA Isolation, Retrotranscription, and Quantitative PCR (qPCR) Analysis

Total cellular RNA was isolated using TRI-Reagent (SIGMA-Aldrich^®^) according to the manufacturer’s instructions. The concentration and purity of the RNA samples were assessed using a NanoDrop^®^ 1000 spectrophotometer (Thermo Fisher, Waltham, MA, USA). Here, 1 μg of RNA was reverse transcribed into cDNA using SuperScript III reverse transcriptase (Invitrogen, Thermo Fisher). qPCR was performed on three independent biological replicates, in technical duplicate for each biological replicate using the SYBR green (Applied Biosystems, Foster City, CA, USA) method and an Applied Biosystems 7500 System. The reaction mixture contained 50 ng of cDNA template and 400 nM of each forward and reverse primer in a final volume of 15 μL. The PCR conditions included a denaturation step (95 °C for 10 min) followed by 40 cycles of amplification and quantification (95 °C for 35 s, 60 °C for 1 min). The relative gene expression levels were normalized to the reference gene Glyceraldehyde-3-phosphate dehydrogenase (GAPDH), the expression of which is not affected under our experimental conditions (Table S1) and calculated by the 2−^ΔΔCt^ method. The sequences of the primers used are listed in Table S2.

### 4.4. Generation and Validation of EGR1 KO Cell Line

The EGR1 knockout in the N-enriched SH-SY5Y and HEK293T cell lines was obtained using the CRISPR/Cas9 procedure reported by Ran and colleagues [39]. The strategy was designed to obtain an INDEL mutation in the ORF (open reading frame) of the gene. Briefly, forward and reverse oligonucleotides (Table S2) for the gRNA were designed using the online CRISPR design tool (http://crispr.mit.edu/) and inserted in the all-in-one vector pSpCas9(BB)-2A-Puro (PX459) V2.0 (#62988, Addgene, Watertown, MA, USA) [39]. Next, the generated vector was transfected into the cells using Lipofectamine 3000 (Thermo Fisher) according to the manufacturer’s instructions. After 24 h of transfection, the cells were cultured under puromycin selection (1 µg mL^−1^) for 48 h. The surviving cells were left to propagate on the plate and then transferred to a 96-well plate for single clone selection by serial dilution. The monoclonal population carrying mutated sequence was selected by sequence analysis of the genomic region of interest. In particular, the genomic DNA (gDNA) from 1 × 10^6^ WT and KO cells was isolated using the Quick-gDNA^TM^ Miniprep kit (ZYMO RESEARCH, Irvine, CA, USA) according to the manufacturer’s instructions, and the region of interest was amplified using a specific primer pair (Table S2). RT-PCR was performed using Taq Master Mix (NEB, Ipswich, MA, USA). The reaction mixture contained 1 ng of gDNA template and 400 nM of each forward and reverse primer in a final volume of 20 μL. The PCR conditions included a denaturation step (95 °C for 2 min) followed by 38 cycles of denaturation, annealing, and elongation (95 °C for 30 s, 60 °C for 40 s, and 68 °C for 1 min). Finally, we validated the frameshift mutation in the KO population by Western blotting analysis and checked the expression of EGR1 protein in WT and KO cell lines after serum stimulation. Cell pellets were lysed in RIPA buffer (50 mM Tris-HCl pH 8.8, 150 mM NaCl, 1 mM EDTA, 0.1% SDS, 1% Triton X-100) containing protease inhibitors (Roche, Basel, Switzerland), incubated on ice for 30 min, and centrifuged at 14,000 rpm for 10 min at 4 °C. The supernatant was collected and used for protein quantification by a Bradford assay (BIO-RAD, Hercules, CA, USA). In each sample, 30 μg of protein lysate was electrophoresed in SDS gel (12% acrylamide) and blotted onto a nitrocellulose membrane. The transferred membrane was blocked with 5% non-fat milk (BIO-RAD) in TBST buffer (100 mM Tris-HCl pH 8, 1.5 M NaCl, 0.1% Tween) for 1 h at room temperature (RT) and incubated with primary antibodies (Table S3) in TBST with 3% non-fat milk (BIO-RAD) overnight at 4 °C. After several washes with TBST, the membrane was incubated with the corresponding secondary antibody (Table S3) in TBST with 3% non-fat milk (BIO-RAD). After several washes, immunoreactive bands were visualized using an ECL detection kit (EuroClone^®^) according to the manufacturer’s instructions, and the signals were detected using the Image Lab software (BIO-RAD).

### 4.5. Statistical Analysis

The results from independent biological replicates are expressed as mean ± SEM. Statistical analysis of the qPCR data was carried out using a two-tailed t test (Prism 6 software) with a *p*-value cut-off of 0.05.

## Supplementary Materials

The following are available online at https://www.mdpi.com/2311-553X/6/4/46/s1, Figure S1: Basic elements of the CRISPR/Cas9 technology used to knockout the EGR1 gene and validation of the EGR1 KO cell line; Table S1: Expression level of the reference gene Glyceraldehyde-3-phosphate dehydrogenase (GAPDH) in different experimental conditions; Table S2: Sequences of gRNAs, genotyping primers and primers used for qPCR analysis; Table S3: Primary and secondary antibodies for western blotting analysis.

## References

[1] Fowler T., Sen R., Roy A.L. (2011). Regulation of Primary Response Genes. Mol. Cell.

[2] Aitken S., Magi S., Alhendi A.M.N., Itoh M., Kawaji H., Lassmann T., Daub C.O., Arner E., Carninci P., Forrest A.R.R. (2015). Transcriptional Dynamics Reveal Critical Roles for Non-coding RNAs in the Immediate-Early Response. PLoS Comput. Biol..

[3] West A.E., Greenberg M.E. (2011). Neuronal Activity-Regulated Gene Transcription in Synapse Development and Cognitive Function. Cold Spring Harb. Perspect. Biol..

[4] Aliperti V., Donizetti A. (2016). Long Non-coding RNA in Neurons: New Players in Early Response to BDNF Stimulation. Front. Mol. Neurosci..

[5] Bunch H., Choe H., Kim J., Jo D.S., Jeon S., Lee S., Cho D.-H., Kang K. (2019). P-TEFb Regulates Transcriptional Activation in Non-coding RNA Genes. Front. Genet..

[6] Bunch H., Lawney B.P., Burkholder A., Ma D., Zheng X., Motola S., Fargo D.C., Levine S.S., Wang Y.E., Hu G. (2016). RNA polymerase II promoter-proximal pausing in mammalian long non-coding genes. Genomics.

[7] Bunch H. (2018). Gene regulation of mammalian long non-coding RNA. Mol. Genet. Genom..

[8] Rahl P.B., Lin C.Y., Seila A.C., Flynn R.A., McCuine S., Burge C.B., Sharp P.A., Young R.A. (2010). c-Myc Regulates Transcriptional Pause Release. Cell.

[9] Zobeck K.L., Buckley M.S., Zipfel W.R., Lis J.T. (2010). Recruitment Timing and Dynamics of Transcription Factors at the Hsp70 Loci in Living Cells. Mol. Cell.

[10] Bunch H. (2017). RNA polymerase II pausing and transcriptional regulation of the HSP70 expression. Eur. J. Cell Biol..

[11] Nie L., Wu H.-J., Hsu J.-M., Chang S.-S., Labaff A.M., Li C.-W., Wang Y., Hsu J.L., Hung M.-C. (2012). Long non-coding RNAs: Versatile master regulators of gene expression and crucial players in cancer. Am. J. Transl. Res..

[12] Ulitsky I., Bartel D.P. (2013). lincRNAs: Genomics, Evolution, and Mechanisms. Cell.

[13] Kung J.T.Y., Colognori D., Lee J.T. (2013). Long Noncoding RNAs: Past, Present, and Future. Genetics.

[14] Quinn J.J., Chang H.Y. (2016). Unique features of long non-coding RNA biogenesis and function. Nat. Rev. Genet..

[15] Zhao Y., Li H., Fang S., Kang Y., Wu W., Hao Y., Li Z., Bu D., Sun N., Zhang M.Q. (2016). NONCODE 2016: An informative and valuable data source of long non-coding RNAs. Nucleic Acids Res..

[16] Derrien T., Johnson R., Bussotti G., Tanzer A., Djebali S., Tilgner H., Guernec G., Martin D., Merkel A., Knowles D.G. (2012). The GENCODE v7 catalog of human long noncoding RNAs: Analysis of their gene structure, evolution, and expression. Genome Res..

[17] Ransohoff J.D., Wei Y., Khavari P.A. (2018). The functions and unique features of long intergenic non-coding RNA. Nat. Rev. Mol. Cell Biol..

[18] Barry G. (2014). Integrating the roles of long and small non-coding RNA in brain function and disease. Mol. Psychiatry.

[19] St Laurent G., Wahlestedt C. (2007). Noncoding RNAs: Couplers of analog and digital information in nervous system function?. Trends Neurosci..

[20] Lin M., Pedrosa E., Shah A., Hrabovsky A., Maqbool S., Zheng D., Lachman H.M. (2011). RNA-Seq of Human Neurons Derived from iPS Cells Reveals Candidate Long Non-Coding RNAs Involved in Neurogenesis and Neuropsychiatric Disorders. PLoS ONE.

[21] Talkowski M.E., Maussion G., Crapper L., Rosenfeld J.A., Blumenthal I., Hanscom C., Chiang C., Lindgren A., Pereira S., Ruderfer D.M. (2012). Disruption of a Large Intergenic Noncoding RNA in Subjects with Neurodevelopmental Disabilities. Am. J. Hum. Genet..

[22] Johnson R. (2012). Long non-coding RNAs in Huntington’s disease neurodegeneration. Neurobiol. Dis..

[23] Lipovich L., Dachet F., Cai J., Bagla S., Balan K., Jia H., Loeb J.A. (2012). Activity-Dependent Human Brain Coding/Noncoding Gene Regulatory Networks. Genetics.

[24] Nishimoto Y., Nakagawa S., Hirose T., Okano H.J., Takao M., Shibata S., Suyama S., Kuwako K.I., Imai T., Murayama S. (2013). The Long Non-Coding RNA Nuclear-Enriched Abundant Transcript 1_2 Induces Paraspeckle Formation in the Motor Neuron during the Early Phase of Amyotrophic Lateral Sclerosis. Mol. Brain.

[25] Petazzi P., Sandoval J., Szczesna K., Jorge O.C., Roa L., Sayols S., Gomez A., Huertas D., Esteller M. (2013). Dysregulation of the long non-coding RNA transcriptome in a Rett syndrome mouse model. RNA Biol..

[26] Ziats M.N., Rennert O.M. (2013). Aberrant Expression of Long Noncoding RNAs in Autistic Brain. J. Mol. Neurosci..

[27] Van de Vondervoort I.G.M., Gordebeke P.M., Ekhoshab N., Tiesinga P.H., Buitelaar J.K., Kozicz T., Aschrafi A., Glennon J.C. (2013). Long non-coding RNAs in neurodevelopmental disorders. Front. Mol. Neurosci..

[28] Wu P., Zuo X., Deng H., Liu X., Liu L., Ji A. (2013). Roles of long noncoding RNAs in brain development, functional diversification and neurodegenerative diseases. Brain Res. Bull..

[29] Roberts T.C., Morris K.V., Woo M.J.A. (2014). The role of long non-coding RNAs in neurodevelopment, brain function and neurological disease. Philos. Trans. R. Soc. B Biol. Sci..

[30] Wei C.-W., Luo T., Zou S.-S., Wu A.-S. (2018). The Role of Long Noncoding RNAs in Central Nervous System and Neurodegenerative Diseases. Front. Behav. Neurosci..

[31] Li L., Zhuang Y., Zhao X., Li X. (2019). Long Non-coding RNA in Neuronal Development and Neurological Disorders. Front. Genet..

[32] Zimmer-Bensch G. (2019). Emerging Roles of Long Non-Coding RNAs as Drivers of Brain Evolution. Cells.

[33] Reitmair A., Sachs G., Bin Im W., Wheeler L., Im W.B. (2012). C6orf176: A novel possible regulator of cAMP-mediated gene expression. Physiol. Genom..

[34] Pruunsild P., Bengtson C.P., Bading H. (2017). Networks of Cultured iPSC-Derived Neurons Reveal the Human Synaptic Activity-Regulated Adaptive Gene Program. Cell Rep..

[35] Issler O., Van Der Zee Y.Y., Ramakrishnan A., Wang J., Tan C., Loh Y.-H.E., Purushothaman I., Walker D.M., Lorsch Z.S., Hamilton P.J. (2020). Sex-Specific Role for the Long Non-coding RNA LINC00473 in Depression. Neuron.

[36] Bahrami S., Drabløs F. (2016). Gene regulation in the immediate-early response process. Adv. Biol. Regul..

[37] Schratt G., Weinhold B., Lundberg A.S., Schuck S., Berger J., Schwarz H., Weinberg R.A., Rüther U., Nordheim A. (2001). Serum Response Factor Is Required for Immediate-Early Gene Activation yet Is Dispensable for Proliferation of Embryonic Stem Cells. Mol. Cell. Biol..

[38] Lee S.-M., Vasishtha M., Prywes R. (2010). Activation and Repression of Cellular Immediate Early Genes by Serum Response Factor Cofactors. J. Biol. Chem..

[39] Ran F.A., Hsu P.D., Wright J., Agarwala V., Scott D.A., Zhang F. (2013). Genome engineering using the CRISPR-Cas9 system. Nat. Protoc..

[40] Duclot F., Kabbaj M. (2017). The Role of Early Growth Response 1 (EGR1) in Brain Plasticity and Neuropsychiatric Disorders. Front. Behav. Neurosci..

[41] Arora S., Wang Y., Jia Z., Vardar-Sengul S., Munawar A., Doctor K.S., Birrer M.J., McClelland M., Adamson E., Mercola D. (2008). Egr1 regulates the coordinated expression of numerous EGF receptor target genes as identified by ChIP-on-chip. Genome Biol..

[42] Gallo F.T., Katche C., Morici J.F., Medina J.H., Weisstaub N.V. (2018). Immediate Early Genes, Memory and Psychiatric Disorders: Focus on c-Fos, Egr1 and Arc. Front. Behav. Neurosci..

[43] Christy B., Nathans D. (1989). DNA Binding Site of the Growth Factor-Inducible Protein Zif268. Proc. Natl. Acad. Sci. USA.

[44] Pavletich N.P., O Pabo C. (1991). Zinc finger-DNA recognition: Crystal structure of a Zif268-DNA complex at 2.1 A. Science.

[45] ENCODE Project Consortium (2012). An Integrated Encyclopedia of DNA Elements in the Human Genome. Nature.

[46] ENCODE Project Consortium (2011). A User’s Guide to the Encyclopedia of DNA Elements (ENCODE). PLoS Biol..

[47] Farré D., Roset R., Huerta M., Adsuara J.E., Roselló L., Albà M.M., Messeguer X. (2003). Identification of patterns in biological sequences at the ALGGEN server: PROMO and MALGEN. Nucleic Acids Res..

[48] Grabe N. (2002). AliBaba2: Context Specific Identification of Transcription Factor Binding Sites. In Silico Biol..

[49] Vaudry D., Stork P.J.S., Lazarovici P., Eiden L.E. (2002). Signaling Pathways for PC12 Cell Differentiation: Making the Right Connections. Science.

[50] Nagashima T., Shimodaira H., Ide K., Nakakuki T., Tani Y., Takahashi K., Yumoto N., Hatakeyama M. (2006). Quantitative Transcriptional Control of ErbB Receptor Signaling Undergoes Graded to Biphasic Response for Cell Differentiation. J. Biol. Chem..

[51] D’Agostino S., Testa M., Aliperti V., Venditti M., Minucci S., Aniello F., Edonizetti A. (2019). Expression pattern dysregulation of stress- and neuronal activity-related genes in response to prenatal stress paradigm in zebrafish larvae. Cell Stress Chaperons.

[52] Tullai J.W., Schaffer M.E., Mullenbrock S., Sholder G., Kasif S., Cooper G.M. (2007). Immediate-Early and Delayed Primary Response Genes Are Distinct in Function and Genomic Architecture. J. Biol. Chem..

[53] Chen Z., Li J.-L., Lin S., Cao C., Gimbrone N.T., Yang R., Fu D.A., Carper M.B., Haura E.B., Schabath M.B. (2016). cAMP/CREB-regulated LINC00473 marks LKB1-inactivated lung cancer and mediates tumor growth. J. Clin. Investig..

[54] Zhang L., Wang Y., Li X., Xia X., Li N., He R., He H., Han C., Zhao W. (2017). ZBTB7A Enhances Osteosarcoma Chemoresistance by Transcriptionally Repressing lncRNALINC00473-IL24 Activity. Neoplasia.

[55] Shi C., Yang Y., Yu J., Meng F., Zhang T., Gao Y. (2017). The long noncoding RNA LINC00473, a target of microRNA 34a, promotes tumorigenesis by inhibiting ILF2 degradation in cervical cancer. Am. J. Cancer Res..

[56] Chen Z., Lin S., Li J.-L., Ni W., Guo R., Lu J., Kaye F.J., Wu L. (2018). CRTC1-MAML2 fusion-induced lncRNA LINC00473 expression maintains the growth and survival of human mucoepidermoid carcinoma cells. Oncogene.

[57] Han P.-B., Ji X.-J., Zhang M., Gao L.-Y. (2018). Upregulation of lncRNA LINC00473 promotes radioresistance of HNSCC cells through activating Wnt/β-catenin signaling pathway. Eur. Rev. Med. Pharmacol. Sci..

[58] Chen H., Yang F., Li X., Gong Z.-J., Wang L.-W. (2018). Long noncoding RNA LNC473 inhibits the ubiquitination of survivin via association with USP9X and enhances cell proliferation and invasion in hepatocellular carcinoma cells. Biochem. Biophys. Res. Commun..

[59] Zhang W., Song Y. (2018). LINC00473 predicts poor prognosis and regulates cell migration and invasion in gastric cancer. Biomed. Pharmacother..

[60] Shi X., Wang X. (2019). LINC00473 mediates cyclin D1 expression through a balance between activation and repression signals in breast cancer cells. FEBS Lett..

[61] Zhou W., Zhang M., Liu C., Kang Y., Wang J., Yang X. (2019). Long noncoding RNA LINC00473 drives the progression of pancreatic cancer via upregulating programmed death-ligand 1 by sponging microRNA-195-5p. J. Cell. Physiol..

[62] Chen W., Zhang Y., Wang H., Pan T., Zhang Y., Li C. (2019). LINC00473/miR-374a-5p regulates esophageal squamous cell carcinoma via targeting SPIN1 to weaken the effect of radiotherapy. J. Cell. Biochem..

[63] Shaw G., Morse S., Ararat M., Graham F.L. (2002). Preferential transformation of human neuronal cells by human adenoviruses and the origin of HEK 293 cells. FASEB J..

[64] Lin Y.-C., Boone M., Meuris L., Lemmens I., Van Roy N., Soete A., Reumers J., Moisse M., Plaisance S., Drmanac R.T. (2014). Genome dynamics of the human embryonic kidney 293 lineage in response to cell biology manipulations. Nat. Commun..

[65] Aliperti V., Sgueglia G., Aniello F., Vitale E., Fucci L., Edonizetti A. (2019). Identification, Characterization, and Regulatory Mechanisms of a Novel EGR1 Splicing Isoform. Int. J. Mol. Sci..

[66] Caspi A., Moffitt T.E. (2006). Gene–environment interactions in psychiatry: Joining forces with neuroscience. Nat. Rev. Neurosci..

[67] Covington H.E., Lobo M.K., Maze I., Vialou V., Hyman J.M., Zaman S., LaPlant Q., Mouzon E., Ghose S., Tamminga C.A. (2010). Antidepressant Effect of Optogenetic Stimulation of the Medial Prefrontal Cortex. J. Neurosci..

[68] Stack A., Carrier N., Dietz D., Hollis F., Sorenson J., Kabbaj M. (2010). Sex Differences in Social Interaction in Rats: Role of the Immediate-Early Gene zif268. Neuropsychopharmacology.

[69] Kimoto S., Bazmi H.H., Lewis D.A. (2014). Lower Expression of Glutamic Acid Decarboxylase 67 in the Prefrontal Cortex in Schizophrenia: Contribution of Altered Regulation by Zif268. Am. J. Psychiatry.

