# Supplementary material for: LINC00473 as an Immediate Early Gene under the Control of the EGR1 Transcription Factor
Source: Noncoding RNA. 2020 Nov 12;6(4):46. doi: 10.3390/ncrna6040046 (PMC7712511; doi:10.3390/ncrna6040046)
Supplement: Supplementary file 1 [file ncrna-06-00046-s001.zip › Table S1.docx]

**Table S1.** Expression level of the reference gene Glyceraldehyde-3-phosphate dehydrogenase (GAPDH) in different experimental conditions (means of biological triplicates).

| Experiment | Time point | Mean ± SEM |
| --- | --- | --- |
| BDNF Stimulation in  N-enriched SH-SY5Y cells  (Figure 1A) | 0h  0.5h  1h  2h  4h | 15.505 ± 0.494  15.309 ± 0.936  15.441 ± 0.757  14.699 ± 0.425  15.741 ± 0.215 |
|  |  |  |
| Serum Stimulation in  N-enriched SH-SY5Y cells  (Figure 1B) | 0h  0.5h  1h  2h  4h | 17.097 ± 0.085  17.454 ± 0.426  17.156 ± 0.384  17.086 ± 0.336  17.202 ± 0.476 |
|  |  |  |
| Serum Stimulation in  HEK293T cells  (Figure 1C) | 0h  0.5h  1h  2h  4h | 14.929 ± 0.338  14.825 ± 0.272  14.759 ± 0.272  14.714 ± 0.252  15.504 ± 0.465 |
|  |  |  |
| Serum Stimulation in  N-enriched SH-SY5Y EGR1-KO cells (Figure 2A) | 0h  2h | 13.793 ± 0.562  13.926 ± 0.441 |
|  |  |  |
| Serum Stimulation in  HEK293T EGR1-KO cells  (Figure 2B) | 0h  2h | 14.777 ± 0.923  14.163 ± 0.776 |
