# Supplementary material for: LINC00473 as an Immediate Early Gene under the Control of the EGR1 Transcription Factor
Source: Noncoding RNA. 2020 Nov 12;6(4):46. doi: 10.3390/ncrna6040046 (PMC7712511; doi:10.3390/ncrna6040046)
Supplement: Supplementary file 1 [file ncrna-06-00046-s001.zip › Table S2.docx]

**Table S2.** Sequences of gRNAs, genotyping primers and primers used for qPCR analysis.

| Name | Sequence |
| --- | --- |
| EGR1 gRNA | TOP_5’- CACCGCTGCAGATCTCTGACCCGTT -3’  BOTTOM_5’- AAACAACGGGTCAGAGATCTGCAGC -3’ |
| EGR1 sequencing primers | F_5’- CCGACACCAGCTCTCCAG -3’  R_5’- CTGCGGTCAGGTGCTCGTAG -3’ |
| GAPDH | F_5’- AAAATCAAGTGGGGCGATGC -3’  R_5’- GGCAGAGATGATGACCCTTT -3’ |
| LINC00473 | F_5’- GTCAGCATACTTTGGCGGAC -3’  R_5’- GTTGGTGCACGTGGGAGT -3’ |
| EGR1 | F_5’- GAGCAGCCCTACGAGCAC -3’  R_5’- GGCCACAAGGTGTTGCCA -3’ |
