# Supplementary material for: LINC00473 as an Immediate Early Gene under the Control of the EGR1 Transcription Factor
Source: Noncoding RNA. 2020 Nov 12;6(4):46. doi: 10.3390/ncrna6040046 (PMC7712511; doi:10.3390/ncrna6040046)
Supplement: Supplementary file 1 [file ncrna-06-00046-s001.zip › Table S3.docx]

**Table S3.** Primary and secondary antibodies for western blotting analysis.

| Antibody | Dilution |
| --- | --- |
| Anti-EGR1 Polyclonal Antibody (Immunobiological Sciences),  rabbit (AB-83620) | 1:1000 |
| Anti-GAPDH Monoclonal Antibody (Immunological Sciences),  mouse (MAB-91903) | 1:5000 |
| Donkey Anti-rabbit IgG Secondary Antibody, HRP Conjugate  (A120-108P) | 1:5000 |
| Goat Anti-mouse IgM Secondary Antibody, HRP Conjugate  (BA1075) | 1:5000 |
