# Supplementary material for: LINC00473 as an Immediate Early Gene under the Control of the EGR1 Transcription Factor
Source: Noncoding RNA. 2020 Nov 12;6(4):46. doi: 10.3390/ncrna6040046 (PMC7712511; doi:10.3390/ncrna6040046)
Supplement: Supplementary file 1 [file ncrna-06-00046-s001.zip › Figure S1. Basic elements of the CRISPR-Cas9 technology.docx]

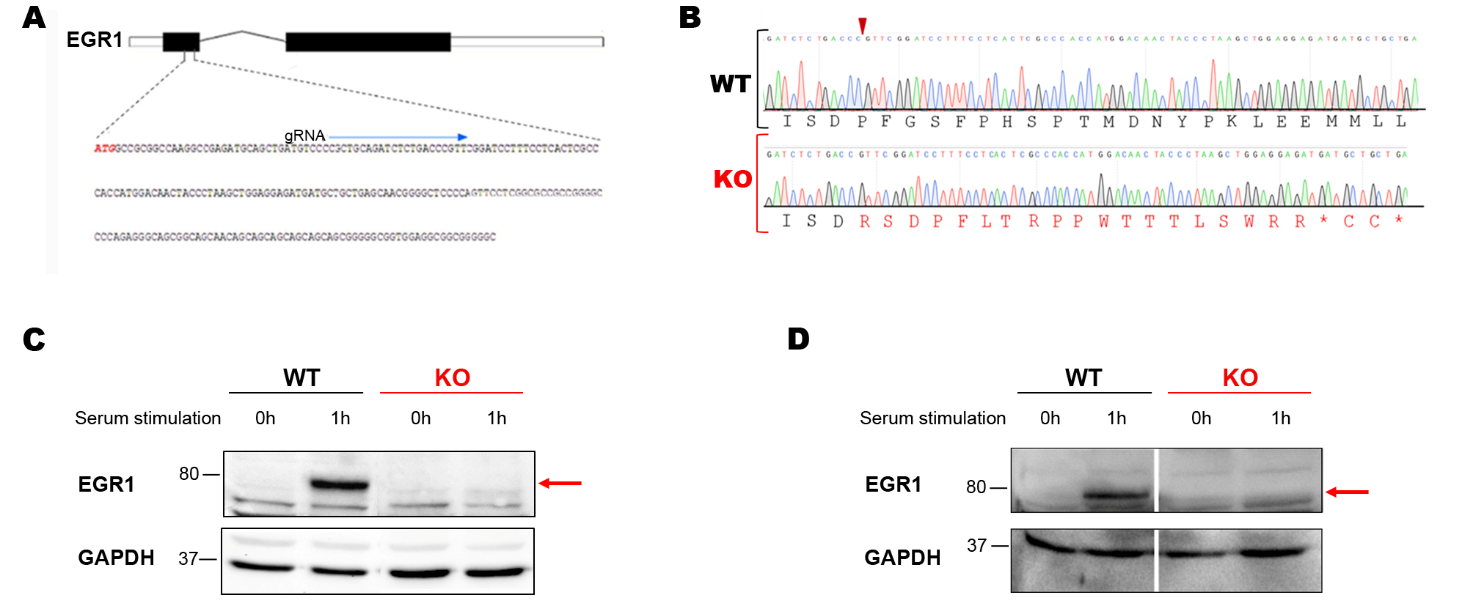


**Figure S1. Basic elements of the CRISPR/Cas9 technology used to knockout the EGR1 gene and validation of the EGR1 KO cell line. A)** Schematic representation of the EGR1 transcript and the corresponding DNA sequence recognized by the gRNA. The rectangles represented the two exons of EGR1 transcript with the black region representing the coding region. The ATG of the EGR1 gene is highlighted in red, while the arrow indicated the gRNA target sequence. **B)** Electropherograms of both WT and KO EGR1 genomic sequence. EGR1 protein level analysed by western blotting at the indicated time points in WT and KO N-enriched SH-SY5Y cells **(C)** and HEK293T cells **(D)**. The level of GAPDH protein was used as a loading control.
